# Sleep Disturbances in Phenylketonuria: An Explorative Study in Men and Mice

**DOI:** 10.3389/fneur.2017.00167

**Published:** 2017-04-26

**Authors:** Vibeke M. Bruinenberg, Marijke C. M. Gordijn, Anita MacDonald, Francjan J. van Spronsen, Eddy A. Van der Zee

**Affiliations:** ^1^Molecular Neurobiology, Groningen Institute for Evolutionary Life Sciences (GELIFES), University of Groningen, Groningen, Netherlands; ^2^Chrono@work B.V., Groningen, Netherlands; ^3^Chronobiology, Groningen Institute for Evolutionary Life Sciences (GELIFES), University of Groningen, Groningen, Netherlands; ^4^Birmingham Children’s Hospital, Birmingham, UK; ^5^Beatrix Children’s Hospital, University Medical Center Groningen, Groningen, Netherlands

**Keywords:** inherited metabolic disorder, Pah^enu2^ mice, phenylketonuria patients, phenylketonuria mice, neurotransmitters, sleep disorders

## Abstract

Sleep problems have not been directly reported in phenylketonuria (PKU). In PKU, the metabolic pathway of phenylalanine is disrupted, which, among others, causes deficits in the neurotransmitters and sleep modulators dopamine, norepinephrine, and serotonin. Understanding sleep problems in PKU patients may help explain the pathophysiology of brain dysfunction in PKU patients. In this explorative study, we investigated possible sleep problems in adult treated PKU patients and untreated PKU mice. In the PKU patients, sleep characteristics were compared to healthy first degree relatives by assessment of sleep disturbances, sleep–wake patterns, and sleepiness with the help of four questionnaires: Holland sleep disorder questionnaire, Pittsburgh sleep quality index, Epworth sleepiness scale, and Munich Chronotype Questionnaire. The results obtained with the questionnaires show that PKU individuals suffer more from sleep disorders, a reduced sleep quality, and an increased latency to fall asleep and experience more sleepiness during the day. In the PKU mice, activity patterns were recorded with passive infrared recorders. PKU mice switched more often between active and non-active behavior and shifted a part of their resting behavior into the active period, confirming that sleep quality is affected as a consequence of PKU. Together, these results give the first indication that sleep problems are present in PKU. More detailed future research will give a better understanding of these problems, which could ultimately result in the improvement of treatment strategies by including sleep quality as an additional treatment target.

## Introduction

In 2014, Gadoth and Oksenberg reviewed the incidence of sleep and sleep disorders in patients with inherited metabolic disease (IMD). Although their review focused on sleep-related breathing disorders among severely affected subjects with IMD, the general title suggested that sleep problems could be missed or underestimated in these conditions. A metabolic disease in which brain modulators of sleep are severely affected but attention for sleep research is very limited is phenylketonuria (PKU). PKU is caused by an inborn error in the metabolic pathway of phenylalanine (Phe) that disrupts the conversion of Phe to tyrosine. As a result, Phe concentrations build up in blood and brain and the ability to intrinsically produce the dopamine precursor tyrosine is lost. These changes do not solely affect the metabolism of dopamine. Also, reduced concentrations of noradrenaline and serotonin are found in PKU patients (1, 2) and in the PKU mouse model (3). These neurotransmitters are known to be important regulators of sleep, wakefulness, and switches between these states (4, 5). Nevertheless, these abnormalities in neurotransmitter availability are not specifically linked to possible sleep problems in PKU research. In PKU research, a few studies have indirectly investigated sleep regulators or sleep. First, in treated and untreated PKU patients, sleep-EEG measurements indicate differences in the number of sleep spindles despite similar REM and non-REM distribution compared to healthy controls (6). Second, in early-treated PKU infants (4–18 weeks old), EEG measurements show differences in the development of sleep compared to healthy controls (7). Finally, in the PKU mouse model, high levels of orexin A (hypocretin 1) were reported, a neuropeptide that is associated with wakefulness (8, 9). This made the authors suggest hyperactivity in PKU, however, the exact consequence of these increased levels are not clear while hyperactivity is not consistently described in PKU mice (10).

Currently, PKU treatment remains suboptimal in which disturbances in executive functions, mood, social cognition, and in internalizing problems, such as depression and anxiety, are described in early-treated PKU patients (11, 12). As it is well established that altered sleep negatively influences cognitive performance, most notably in the domains of executive functioning (13–15), and mood by impacting feelings of depression, anxiety, and stress (16, 17), sleep-related issues could very well serve as an explanation of the PKU brain dysfunction despite diet and drug treatment.

Understanding the presence and severity of sleep problems in PKU patients and its pathophysiology could ultimately result in the improvement of treatment strategies by including sleep quality as an additional treatment target. Therefore, the aim of this explorative study was to investigate the presence of sleep disturbances in PKU patients with questionnaires together with analyses of rest/wake patterns in PKU mice, indirectly reflecting sleep characteristics which could confirm the PKU-specific nature of putative sleep issues in PKU patients. As sleep is influenced by, among others, genetic factors (18–21), first-degree relatives (FDR) of PKU patients and wild-type (WT) littermates of each genetic strain of the PKU mouse model were used as controls.

## Materials and Methods

### PKU Patients

#### Subjects

In the summer of 2016, participants for this study were recruited by distributing a link to an electronic survey to subjects associated with the Dutch PKU patient organization. PKU patients and FDR, who did not do shift work in the past 3 months, were asked to fill out four questionnaires with 10 additional questions (date of birth, zip code, gender, height, bodyweight, PKU or control, treatment of PKU, other health issues, smoking, and the use of sleep-promoting drugs). All participants were informed about the scientific purpose of the study and agreed to participate. They completed the questionnaires completely anonymous. To ensure that the questionnaires were not completed by the same individuals more than once, the submissions were checked for uniqueness, focusing on date of birth and IP address. In total, 47 subjects, 25 PKU patients and 23 controls, participated.

#### Sleep Questionnaires

Four validated questionnaires were included in the survey: (1) Holland Sleep Disorders Questionnaire (HSDQ) (22); 40 items, (2) Pittsburgh Sleep Quality Index (PSQI) (23); 19 items, (3) Epworth Sleepiness Questionnaire (ESS) (24); 8 items, and (4) Munich Chronotype Questionnaire (MCTQ) (25); 16 items. First, the HSDQ gives a general score to identify the possible occurrence of a sleep disorder and can differentiate between six main categories of sleep disorders [insomnia, parasomnia, circadian rhythm sleep disorders (CRSD), hypersomnia, sleep-related movement disorders, such as for instance restless legs syndrome, and sleep-related breathing disorder] (22). Second, for the PSQI, seven component scores can be derived (subjective sleep quality, sleep latency, sleep duration, habitual sleep efficiency, sleep disturbances, use of sleep medication, and daytime dysfunction) and computed to a global score (23). Third, the ESS is used to examine sleepiness during the day (for instance during reading) (24). Finally, the MCTQ was used to identify chronotype (the preferred timing of sleep) calculated by taking the mid-point of sleep on free days corrected for the sleep debt acquired during working days (26).

### PKU Mouse Study

To investigate the rest/wake pattern in PKU mice, the home-cage activity of adult (4–7 months) WT and PKU mice of BTBR and C57Bl/6 (B6) background was monitored. Both genetic strains of the PKU mouse model have a point mutation in the gene encoding for phenylalanine hydroxylase causing Phe to rise in blood and brain (10). The PKU model was originally described for the BTBR background but the model was latter crossed back on to the C57Bl/6J background. Currently, both genetic strains are used in PKU research. In-house heterozygous mating pairs were used to breed the following groups of mice: BTBT WT, BTBR PKU, B6 WT, and B6 PKU. These mice were weaned on postnatal day 28, and genotype was established with quantitative PCR (10). The experiment consisted of a habituation phase and a data-acquiring phase. After a 7-day habituation phase, the activity of individual housed mice (cage: 33 cm × 15 cm × 14 cm with nesting material and paper role) were monitored for 7 days with passive infrared detectors (PIR). During the whole experiment, animals were on a 12/12 light/dark cycle and had *ad libitum* access to water and normal chow (RMH-B 2181, ABdiets, Phe: 8.7 g/kg). Data were analyzed with ACTOVIEW [made in-house, described in Ref. (27)]. This program calculated the average daily activity, diurnality [(Sum activity light phase − sum activity dark phase)/total activity], and fragmentation (28) from the files produced by the circadian activity monitor system (CAMS) collected from the PIR.

### Statistics

The statistical analysis was executed with the statistical software IBM SPSS Statistics for Windows, Version 22.0 (Armonk, NY, USA: IBM Corp.). Within this program, the Shapiro–Wilk was used to test normality of the data. The activity of the mice was normally distributed, and a multi-variate ANOVA was used to test the factors group and gender of overall activity, fragmentation, and diurnality. Differences in group characteristics in the patient study were not normally distributed and, therefore, tested with the non-parametric Mann–Whitney *U* test. The chronotype score of the MCTQ, normally distributed, was tested with a univariate ANOVA for group. Age and gender were included as cofactors. The ordinal nature of the HSDQ, PSQI, and ESS scores, made it not possible to test confounding factors parametrically. Therefore, gender and age were explored with generalized linear models, using an ordinal model. Differences in the frequency of occurrence of sleep disorders were also tested with a generalized linear model, using a binary model. Non-parametric Levene’s test was used to test the homogeneity of the data.

## Results

### PKU Patients

#### Subjects

This study is a pilot study serving as a proof-of-concept for sleep-related issues due to PKU. For this reason, individuals were included when they correctly filled out at least one of the questionnaires. Not all questionnaires were correctly filled out by all subjects, which resulted in different group characteristics for each questionnaire (Table 1). For all questionnaire responses, the FDR controls were significantly older than the PKU subjects [HSDQ: *p* < 0.05, PSQI: *p* < 0.05, Epworth Sleepiness Questionnaire (ESS): *p* < 0.05, MCTQ: *p* < 0.05] but no differences were found in BMI (HSDQ: *p* = 0.68, PSQI: *p* = 0.92, ESS: *p* = 0.99, MCTQ: *p* = 0.90). Furthermore, no significant differences were found in gender distribution of the groups [HSDQ: *t*(44) = −0.603, *p* = 0.55, PSQI: *t*(35) = 0.172, *p* = 0.87, ESS: *t*(37) = 0.143, *p* = 0.89, MCTQ: *t*(39) = −0.260, *p* = 0.80]. One FDR control was excluded because she used several sleep-promoting drugs (Trazodon and zolpidem tartrate).

**Table 1:** **Subject characteristics**.

	HSDQ		PSQI		ESS		MCTQ	
	PKU	FDR-control	PKU	FDR-control	PKU	FDR-control	PKU	FDR-control
Number	25	21	21	15	22	17	24	17
Age	29.2 ± 8.8	44.2 ± 10.1	30.1 ± 9.0	46.6 ± 10	29.6 ± 9.1	45.9 ± 10.1	29.0 ± 8.9	45.9 ± 10.1
BMI	24.1 ± 4.3	25.0 ± 5.1	24.5 ± 4.3	24.8 ± 5.6	24.6 ± 4.2	24.6 ± 4.2	24.4 ± 4.1	24.6 ± 4.2
Gender (F/M)	17/8	16/5	15/6	11/4	16/6	12/5	16/8	12/5
Smoking	4	1	4	1	4	1	4	1
Heath issues	5	0	4	0	4	0	5	0
Sleep-promoting drugs	5	0	5	0	5	0	5	0
Kuvan^®^	3		2		2		3	
Kuvan^®^ + protein restricted	4		3		3		3	
Protein restricted	22		16		17		18	

*Data are presented as mean ± SD*.

*F, female; M, male; HSDQ, Holland Sleep Disorders Questionnaire; PSQI, Pittsburgh Sleep Quality Index; ESS, Epworth Sleepiness Questionnaire; MCTQ, Munich Chronotype Questionnaire; PKU, phenylketonuria; FDR-control, first-degree relatives-control*.

#### Frequency of Sleep Disorders

**Figure 1:**
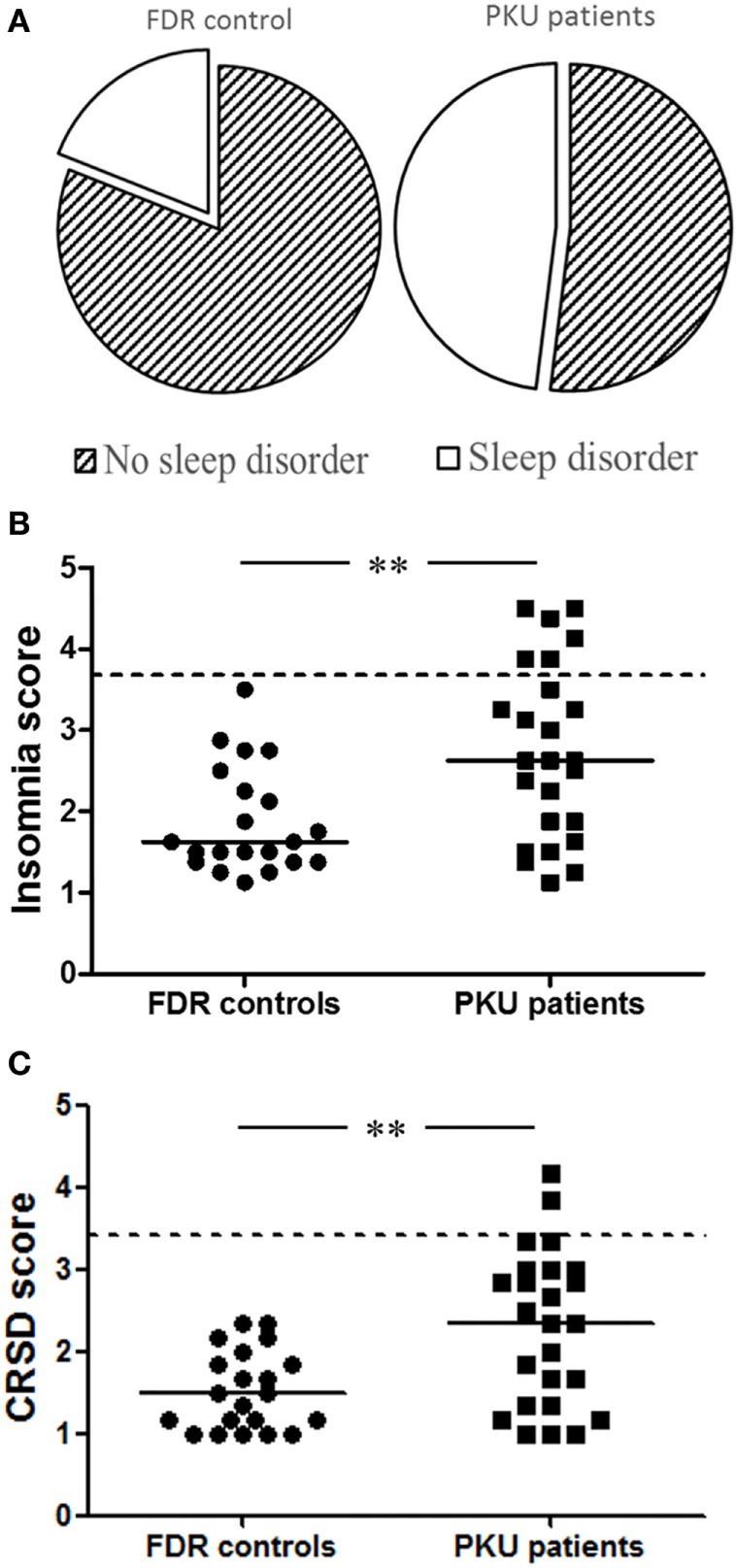
**Holland Sleep Disorders Questionnaire (HSDQ)**. **(A)** Results from the global score of HSDQ indicate that 48% of the phenylketonuria (PKU) patients have a sleep disorder compared to 19% of the first-degree relatives (FDR) controls. **(B)** PKU patients have a significant higher insomnia score than FDR controls. Six PKU patients are above the cutoff score compared to 0 FDR controls. **(C)** Although only two PKU patients are above the cutoff score, PKU patients have significant higher circadian rhythm sleep disorders score compared to FDR controls. Data represent individual scores with median. Dotted line represents cutoff score between having sleep problem or not (**p < 0.01).

The global score of the HSDQ is used to identify the presence of a sleep disorder with an overall accuracy of 88% (κ: 0.75) (22). In PKU patients, 48% had a global score above the cutoff of 2.02, indicative of a sleep disorder, compared to 19% of FDR controls [Figure 1A; *b* = 1.367, Wald χ^2^(1, *N* = 46) = 3.983, *p* < 0.05]. Especially, among the six main sleep disorder categories, PKU patients had a higher score for both insomnia and CRSD [Figure 1B: *b* = −1.527, Wald χ^2^(1, *N* = 46) = 7.626, *p* < 0.05, Figure 1C: *b* = −1.593, Wald χ^2^(1, *N* = 46) = 8.115, *p* < 0.05, respectively], but not for the other four categories [parasomnia; *b* = −0.985, Wald χ^2^(1, *N* = 46) = 2.941, *p* = 0.086, hypersomnia; *b* = −0.985, Wald χ^2^(1, *N* = 46) = 3.287, *p* = 0.070, restless legs syndrome; *b* = −0.696, Wald χ^2^(1, *N* = 46) = 1.757, *p* = 0.185, and sleep-related breathing disorder; *b* = −0.693, Wald χ^2^(1, *N* = 46) = 1.688, *p* = 0.194]. Age and gender did not significantly contribute to these models. These results reveal a higher frequency of sleep disorders, more specifically insomnia and CRSD, in PKU patients.

#### Sleep Quality

**Figure 2:**
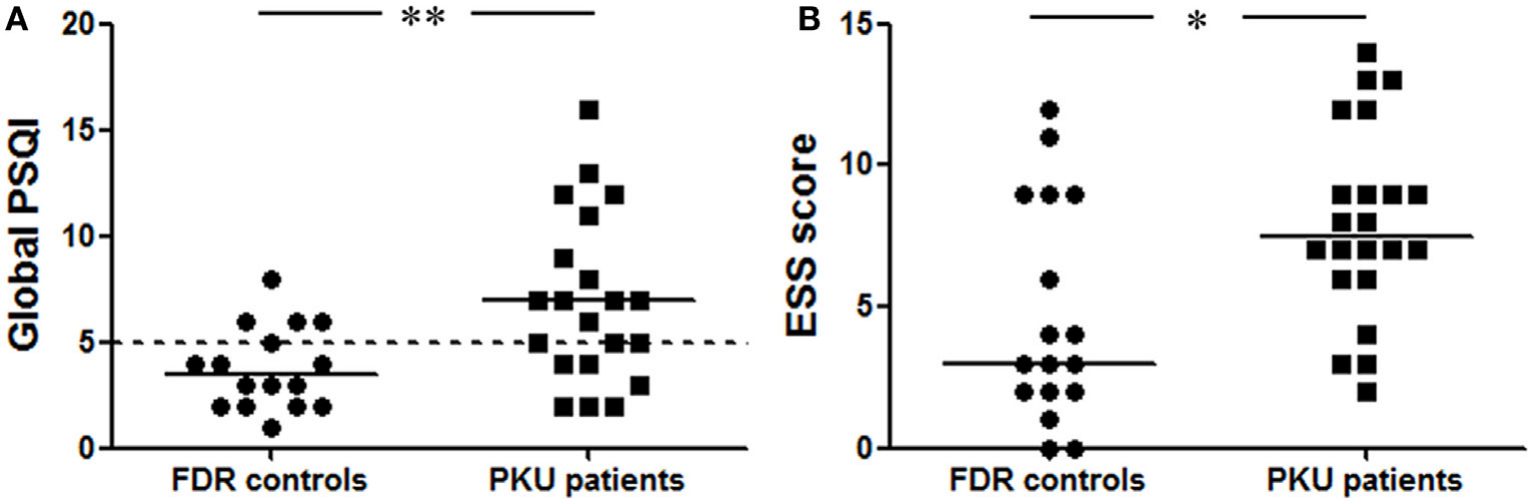
**Pittsburgh Sleep Quality Index (PSQI) and ESS**. **(A)** The global score of the PSQI was significantly higher in phenylketonuria (PKU) patients compared to first-degree relatives (FDR) control. The dotted line represents the cutoff between good and poor sleepers. Fifty-seven percent of the PKU patients are above this cutoff and categorized as poor sleepers. **(B)** Epworth Sleepiness Questionnaire (ESS) patients experience more sleepiness during the day than FDR controls. Data represent individual scores with median (*p < 0.05, **p < 0.01).

The global PSQI, comprised seven sleep-related components, is used to classify poor (>5) and good sleepers (≤5). This global score was significantly higher in the PKU patients of which more people were classified as poor sleepers (57%) compared to the FDR controls of which 25% were classified as poor sleepers [Figure 2A: *b* = −1.674, Wald χ^2^(1, *N* = 37) = 7.102, *p* < 0.05]. Within the global score, two component scores differed between PKU patients and FDR controls. The first was the component score for latency to fall asleep which was significantly increased in PKU patients [FDR: 0.44 ± 0.63, PKU: 1.43 ± 0.98, *b* = −2.306, Wald χ^2^(1, *N* = 37) = 9.733, *p* < 0.05]. The second one was the component score for subjective sleep quality. This score was computed from the question in which subjects could indicate their quality of sleep during the past month on a scale of very good (score 0) to very bad (score 3). This score was significantly higher in PKU patients than in the FDR controls [FDR: 0.69 ± 0.60, PKU: 1.38 ± 0.92, *b* = −1.655, Wald χ^2^(1, *N* = 37) = 5.516, *p* < 0.05]. Age and gender did not significantly contribute to these models. These results suggest that sleep quality is reduced in PKU patients compared to FDR controls.

#### Sleepiness during the Day

In the ESS, the participant subjectively rate the chance of dozing off during eight situations from “none” (score 0) to “high” (score 3). This score is significantly higher in PKU patients than FDR controls [Figure 2B; *b* = −1.608, Wald χ^2^(1, *N* = 39) = 6.597, *p* < 0.05]. A main effect of age and gender did not contribute significantly to the model. These results suggest that PKU patients experience more sleepiness during daytime.

#### Chronotype

In the MCTQ, the sleep schedules of the participants on working and non-working days were asked. From these data, we could calculate chronotype, defined as the mid-sleep on free days corrected for the potential sleep debt acquired during the working days (26). Chronotype is dependent on age and gender (26), therefore, statistical analysis included age and gender as a cofactor. No significant differences were found between chronotype scores in PKU patients and FDR controls or for any of the cofactors in the complete model [group: *F*(1,37) = 1.287, *p* = 0.26, gender: *F*(1,37) = 0.954, *p* = 0.34, age: *F*(1,37) = 1.941, *p* = 0.17].

### PKU Mice

#### Rest/Wake Patterns

**Figure 3:**
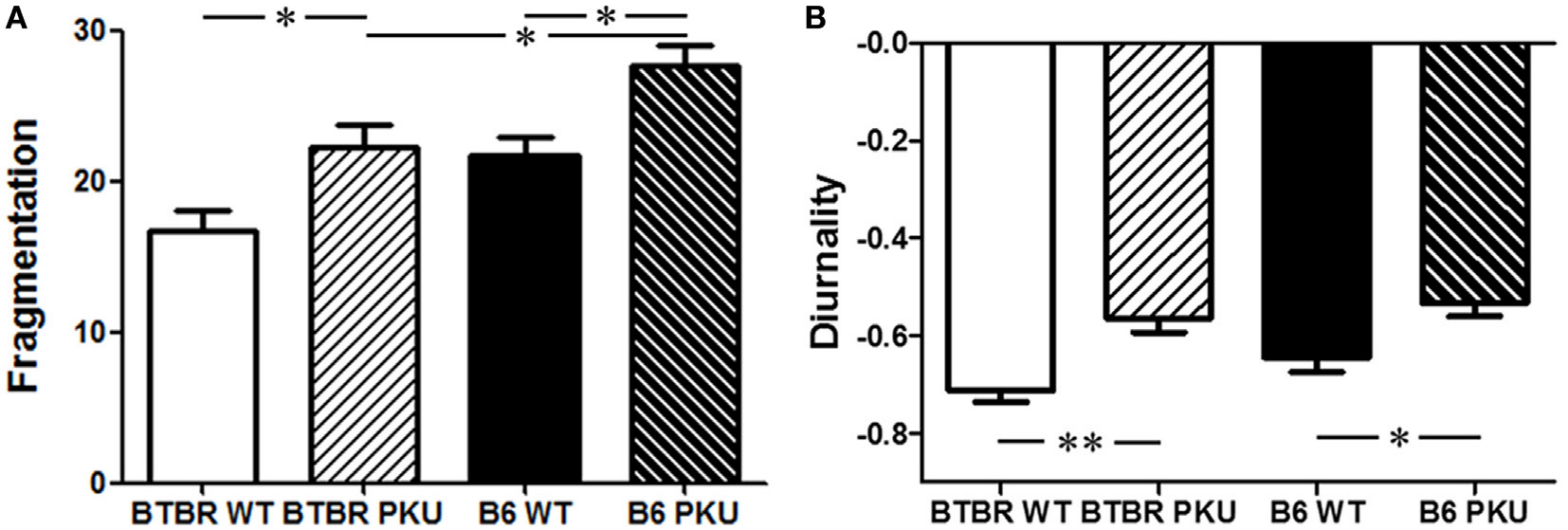
**Characteristics of the rest/wake pattern in mice**. **(A)** In both genetic strains of the phenylketonuria (PKU) mouse model, an increase in fragmentation is seen in the PKU mice compared to WT littermates. Furthermore, a significant difference is found between the fragmentation score of PKU mice of each strain. **(B)** In the graph, negative diurnality scores are observed. This indicates that we are investigating animals which are active in the dark. PKU mice have a less distinct negative score suggesting that they shift part of their resting behavior into the light phase. Data are depicted as mean ± SEM (*p < 0.05, **p < 0.01).

No main or interaction effects were observed for sex in all parameters, therefore, data of males and females were grouped. The fragmentation score is indicative of the frequency that active behavior is switched to non-active behavior and *vice versa*. In PKU mice, in both strains, the fragmentation score was increased compared to WT [Figure 3A; *F*(3,56) = 10.803, *p* < 0.05, BTBR *p* < 0.05, *p* < 0.05]. Although differences were found in overall activity between the WT’s of each strain [BTBR WT: 3,162.46 ± 1,239.24, B6 WT: 2,193.69 ± 785.91 (mean ± SD) *F*(3,56) = 3.858, *p* < 0.05, BTBR WT vs. B6 WT *p* = 0.019], the increase in fragmentation score found in PKU mice did not coincide with a change in overall activity (BTBR PKU: 2,655.42 ± 605.23, B6 PKU: 2,306.90 ± 860.06, BTBR *p* = 0.67, B6 *p* = 1.000). However, a shift did occur in the timing of the rest/active behavior. The negative diurnality score, reflecting night activity in animals, became less negative in PKU mice [Figure 3B; *F*(3,56) = 8.235, *p* < 0.001, BTBR *p* < 0.05, B6 *p* < 0.05]. These results reveal that PKU mice have increased fragmentation and a shift in diurnality (more inactive in active phase).

## Discussion

In this explorative study, we investigated sleep characteristics in PKU patients with questionnaires and analyzed the rest/wake patterns in PKU mice. In the PKU patients study, we showed that PKU patients compared to FDR controls have more sleep disorders, a reduced sleep quality, an increased latency to fall asleep, and experience more sleepiness during the day. In the PKU mice, we found an increase in fragmentation and a shift in diurnality. The increase in fragmentation indicated that the PKU mice switch more often between active and non-active behavior. This score did not coincide with changes in overall activity. In addition, PKU mice shift a part from their resting behavior into the active phase (a shift in diurnality). Both experiments strongly support the hypothesis that sleep is affected in PKU. This seems to be directly related to the disorders as the deficits were found in both PKU mouse strains despite their genetic differences and cognitive sensitivity to the PKU condition (10).

### Study Limitations

Sleep is influenced by, among others, genetic factors, age, and gender (18–21). For this reason, this study recruited FDR of PKU patients as control group. No differences were found in gender distributions between the groups, but differences were found in age between FDR controls and PKU patients. Although we recognize these limitations, we believe that it does not hamper our results obtained in the first three questionnaires (HSDQ, PSQI, ESS) for the following reason. Literature shows either no effect or a deterioration of sleep with aging. For example, the PSQI is not affected by age (29). The ESS score is influenced by an age × gender interaction, wherein females tend to have higher scores between 0 and 39 age group compared to males (30). Around the ages 40–49, the ESS score of males deteriorates reaching the same ESS score as females. Therefore, the ESS score is either higher or stays constant with increasing age (30). This implies that the higher scores found in the younger PKU patients compared to the older FDR controls are contrary to what would be expected and likely reflect a real indication of sleep problems in PKU patients. Moreover, no significant effect of age was found in our study.

### Sleep Characteristics Are Altered in PKU

The different measurements of sleep used in this study reveal a variety of sleep problems and show some specifically affected characteristics of sleep. In the HSDQ, PKU patients show a higher incidence in sleep disorders, but only in the main categories insomnia and CRSD scores were higher in PKU patients than in FDR controls. CRSD were changed to circadian rhythm sleep–wake disorders (CRSWD) in the third edition of the International classification of sleep disorders (31). CRSWD are sleep disorders grouped under dyssomnias, a group of sleep disorders, which show insomnia, excessive sleepiness, or difficulty initiating or maintaining sleep (31). In the current study, we found an increased score for insomnia and an increased sleepiness during the day in the ESS in PKU patients, supporting the idea that PKU patients experience problems specific for this cluster of sleep disorders. CRSWD are disorders related to the timing of sleep (31). Some are a consequence of external circumstances, e.g., shift work or jet lag, others have potentially a more internal, neurological basis, e.g., delayed sleep phase syndrome (DSPS). In DSPS, the latency to fall asleep opposed to the desired time to fall asleep is delayed. DSPS patients experience difficulties to shift their sleep/wake pattern to an earlier time point in response to environmental time cues, for example traveling from Europe to Asia, and do not experience sleepiness when they are able to sleep at their desired time, as is possible for instance during a holiday or vacation period. In this study, we showed that PKU patients report an increased latency to fall asleep in the PSQI. However, we did not see a difference in the mid-sleep on non-working days when age was a cofactor. This could be because mid-sleep is strongly influenced by age and the age was not distributed evenly over the full width of both groups (26). Therefore, an important future direction is to compare age-matched controls to PKU patients. Further research in PKU should focus on (1) more objective measurements of sleep, such as polysomnography or sleep–wake rhythm analysis with (2) phase shift experiments in PKU mice to identify problems in shifting sleep/wake patterns (and neurological substrates), (3) monitoring sleepiness during the day specifically during holidays and (4) core body temperature and dim-light melatonin rhythm monitoring to investigate if PKU patients experience a blunted or delayed internal rhythm of physiological markers.

### The Switch between Sleep and Wakefulness Is Defective in PKU

The HSDQ identifies sleep disorders and attribute certain scores to six symptom clusters. These clusters may be due to different sleep disorders, possibly with different pathophysiological mechanism. For instance, the comorbidity of insomnia with other sleep disorders is very high (22). The PKU mouse study did identify a more specific aspect of sleep, namely increased fragmentation. In general, an increased fragmentation score indicates an increase in switching behavior. Switching between sleep and wakefulness is thought to be regulated by a flip-flop switch that results from mutual inhibition of sleep-promoting pathways and wake-promoting pathways (32). Several cholinergic and monoaminergic projections are important in these pathways, such as serotonin, norepinephrine, and dopamine (32). As these latter neurotransmitters are affected in PKU mice (and in untreated PKU patients), it could be that the increased fragmentation score is a consequence of disruptions in this switch. In early-treated patients on diet, neurotransmitter deficiencies in dopamine and serotonin are still present (2). These deficiencies could possibly affect the switch between sleep and wakefulness and cause fragmentation of the sleep/wake rhythm in PKU patients, more difficulty falling asleep and as a consequence daytime sleepiness.

### Conclusion

This explorative study is the first to investigate sleep disturbances both in PKU patients and PKU mice. In PKU patients, we demonstrate more sleep disorders, a reduced sleep quality, an increased latency to fall asleep, and more sleepiness during the day. We show in PKU mice an increased fragmentation and a shift in diurnality. These results produce the first evidence to suggest that sleep problems occur in PKU. The resulting complaints associated with altered sleep are comparable to the cognitive symptoms described for early and continuously treated PKU patients. More detailed future research will give a better understanding and further identify sleep problems in PKU, which could ultimately result in the improvement of treatment strategies by including sleep quality as an additional treatment target.

## Ethics Statement

The Medical Ethics Review Committee of the University of Groningen concluded that the Medical Research Involving Subjects Act did not apply to this study. All proceedings concerning the mouse study were carried out in accordance to the Guide for the Care and Use of Laboratory Animals of the National Institutes of Health (The ARRIVE Guidelines Checklist) approved by the Institutional Animal Care and Use Committee of the University of Groningen.

## Author Contributions

For the patient study, all authors designed the study. MG made the electronic survey. Data analysis was performed by MG and VB. For the mouse study, EZ and VB designed the study. VB performed the study and the analysis of the data. All authors contributed substantially to the interpretation of the data and drafting the manuscript.

## Conflict of Interest Statement

VB has received grant funding from Nutricia MG is CEO of Chrono@work. A company specialized in sleep research. In addition, this author has received research funding from Philips. AM has received research funding and honoraria from Nutricia, Vitaflo International, Merck Serono, chaired/chairs the European Nutrition Expert Panel (Merck Serono international and later Biomarin), was/is a member of the Sapropterin Advisory Board (Merck Serono international, Biomarin), and is a member of the Advisory Boards Element (Danone-Nutricia) and Arla Foods International. FS is a member of various Scientific Advisory Boards of (in past Merck Serono,) Biomarin, Applied Pharma Research, ARLA Food, and Danone and has received grants for research purpose of both Merck Serono, Biomarin, Sobi and Nutricia. Furthermore, he has received consultation fees and speaker relation fees from Merck Serono, Biomarin, Nutricia, and Vitaflo. He has assisted in the design of clinical studies using products manufactured by Biomarin. He chairs the Scientific Advisory Board of the Dutch and the European PKU society and is the chairman of the group developing guidelines on PKU in Europe. EZ has received grant funding from Nutricia and awards from NPKUA All authors confirm independence from the sponsors; the content of the article has not been influenced by the sponsors. The remaining authors declare that the research was conducted in the absence of any commercial or financial relationships that could be construed as a potential conflict of interest.
